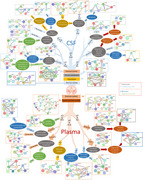# Large‐scale CSF and plasma proteomics reveal dysregulation of immune system, synaptic impairment, and nuclear envelope related pathways in neurodegeneration

**DOI:** 10.1002/alz70856_099428

**Published:** 2025-12-24

**Authors:** Muhammad Ali, Ying Xu, Gyujin Heo, Jigyasha Timsina, Menghan Liu, John P. Budde, Jen Gentsch, Carlos Cruchaga

**Affiliations:** ^1^ Department of Psychiatry, Washington University School of Medicine, St. Louis, MO, USA; ^2^ NeuroGenomics and Informatics Center, Washington University School of Medicine, St. Louis, MO, USA; ^3^ Hope Center for Neurological Disorders, Washington University in St. Louis, St. Louis, MO, USA; ^4^ The Charles F. and Joanne Knight Alzheimer Disease Research Center, St Louis, MO, USA; ^5^ Washington University School of Medicine, St. Louis, MO, USA

## Abstract

**Background:**

Neurodegenerative diseases (NDs) such as Alzheimer's disease (AD), Parkinson's disease (PD), frontotemporal dementia (FTD), and dementia with Lewy bodies (DLB) are characterized by progressive cognitive and motor impairments. Despite substantial efforts, the accurate differentiation between these diseases remains challenging due to overlapping clinical features. Proteomics offers a powerful approach for identifying disease‐specific molecular signatures that can serve as biomarkers for diagnosis, disease classification, and understanding pathophysiological mechanisms.

**Method:**

We generated Somascan (6,500 analytes) in 2,705 CSF and 3,009 plasma samples (CSF/Plasma: AD=1157/1123, PD=739/779, DLB=37/122, FTD=46/42, and controls=726/943). Proteins associated with each disease (FDR < 0.05) were validated in external studies and utilized for developing prediction models as well as performing cell type‐ and pathway‐enrichment analyses. We analyzed disease‐specific biological processes, highlighting protein links to the vulnerability of brain regions uniquely affected in each disease.

**Result:**

We identified disease‐specific proteins in both tissues, with a significantly larger number of associated proteins detected in CSF compared to plasma (CSF/Plasma; AD=4,425/845, PD=370/154, DLB=2,862/148, FTD=3,873/0), suggesting distinct tissue‐specific proteomic alterations across NDs. We demonstrate that AD and DLB are more similar across tissues (CSF/Plasma r^2^=0.59/0.77), while DLB and FTD exhibit stronger CSF‐specific similarity (r^2^=0.89). We developed prediction models specific for AD, PD, FTD, and DLB with high accuracy in CSF (AUC 0.81–0.95) and plasma (AUC 0.8–0.89). Pathway enrichment analyses uncovered exclusive and overlapping pathways driven by unique disease‐specific proteins (Figure 1). Immune system related pathways were commonly dysregulated across NDs in both tissues. The extracellular matrix organization and synaptic impairment related pathways were enriched in AD. Unique to PD were ATF4 activation in response to ER stress and PERK‐regulated gene expression, which contribute to dopaminergic neurons death. DLB included chemokine‐mediated microglial activation and apoptotic pathways, decreasing cell survival in amygdala and basal ganglia. FTD exhibited prominence in nuclear envelope reassembly and interferon signaling.

**Conclusion:**

We delineated both shared and unique molecular pathways across NDs. While each disease expresses unique molecular signatures, converging pathways—e.g. neuroinflammation, synaptic plasticity, and cellular homeostasis—underscore common neurodegenerative processes disrupted by different molecular drivers. These insights can guide targeted therapeutic strategies, enhancing precision medicine for neurodegeneration.